# Alda-1 Ameliorates Liver Ischemia-Reperfusion Injury by Activating Aldehyde Dehydrogenase 2 and Enhancing Autophagy in Mice

**DOI:** 10.1155/2018/9807139

**Published:** 2018-12-24

**Authors:** Meng Li, Min Xu, Jichang Li, Lili Chen, Dongwei Xu, Ying Tong, Jianjun Zhang, Hailong Wu, Xiaoni Kong, Qiang Xia

**Affiliations:** ^1^Department of Liver Surgery, Renji Hospital, School of Medicine, Shanghai Jiao Tong University, Shanghai, China; ^2^Shanghai Key Laboratory for Molecular Imaging, Collaborative Research Center, Shanghai University of Medicine & Health Science, Shanghai, China

## Abstract

Aldehyde dehydrogenase 2 (ALDH2) is a key enzyme for metabolism of reactive aldehydes, but its role during liver ischemia-reperfusion injury (IRI) remains unclear. In the present study, we investigated the effects of the ALDH2 activator, Alda-1, in liver IRI and elucidated the underlying mechanisms. Mice were pretreated with Alda-1 and subjected to a 90 min hepatic 70% ischemia model, and liver tissues or serum samples were collected at indicated time points after reperfusion. We demonstrated that Alda-1 pretreatment had a hepatoprotective role in liver IRI as evidenced by decreased liver necrotic areas, serum ALT/AST levels, and liver inflammatory responses. Mechanistically, Alda-1 treatment enhanced ALDH2 activity and subsequently reduced the accumulation of reactive aldehydes and toxic protein adducts, which result in decreased hepatocyte apoptosis and mitochondrial dysfunction. We further demonstrated that Alda-1 treatment could activate AMPK and autophagy and that AMPK activation was required for Alda-1-mediated autophagy enhancement. These findings collectively indicate that Alda-1-mediated ALDH2 activation could be a promising strategy to improve liver IRI by clearance of reactive aldehydes and enhancement of autophagy.

## 1. Introduction

Liver ischemia-reperfusion injury (IRI) is an ineluctable pathological process during a series of clinical procedures, such as liver transplantation, liver resection, and hemorrhagic shock [1]. Although liver IRI accounts for up to 10% of early graft failure and leads to a higher prevalence of acute or chronic rejection after liver transplantation, no effective pharmacological interventions have been developed to protect the liver from IRI so far [2]. Hepatocyte death and the subsequent inflammatory response are two key features of liver IRI, which form a vicious pathogenesis circle to drive the progression of liver IRI [3]. Therefore, strategies to effectively ameliorate hepatocyte death and interrupt the following inflammatory cascade reactions are urgently needed to improve liver IRI.

Autophagy is widely recognized as a critical protective cellular pathway in response to multiple intra- or extracellular stresses [4]. It has been reported that autophagy is deeply involved in various liver diseases including metabolic diseases, infectious diseases, and cancer [5]. However, the defined function of autophagy in the pathogenesis of IRI remains controversial [6]. We and other groups have demonstrated that the activation of autophagy plays a protective role in liver IRI [7–9].

Oxidative stress is one of the detrimental factors in the pathogenesis of liver IRI and accounts for the major reason of hepatocyte death [10, 11]. As such, excessive ROS could cause the accumulation of reactive aldehydes, including 4-hydroxy-2-nonenal (4HNE) and malondialdehyde (MDA), by lipid peroxidation [12], which can directly attack cellular proteins to form toxic protein to further aggravate liver IRI [13]. Mitochondrial aldehyde dehydrogenase 2 (ALDH2) is a key enzyme responsible for detoxification of those reactive aldehydes to carboxylic acids [14]. Previous studies have indicated that ALDH2 activity is significantly decreased parallel with the remarkable accumulation of reactive aldehydes during liver IRI [15], suggesting that ALDH2 activation may play a protective role in liver IRI through cleaning up toxic aldehydes. Alda-1, a well-characterized ALDH2 activator, serves to activate or restore ALDH2 catalytic activity by modifying the kinetic properties of ALDH2 and increasing the substrate-enzyme interaction [16–18]. Previous studies have demonstrated that Alda-1 treatment significantly improves IRI in various types of organs including the heart, brain, lung, kidney, and intestine [16, 19–22]. However, whether Alda-1 plays a protective role in liver IRI remains unknown.

Here, we adopted an in vivo mouse liver IRI model together with an *in vitro* primary hepatocyte hypoxia/reoxygenation (H/R) injury model to investigate whether Alda-1 plays a protective role in liver IRI by activating ALDH2-mediated cleanup of reactive aldehydes. We found that Alda-1-induced pharmacological activation of ALDH2 increased the clearance of reactive aldehydes, enhanced hepatic autophagy, and ameliorated liver IRI. Thus, we claim that Alda-1 treatment may have clinical implications to protect against liver IRI.

## 2. Materials and Methods

### 2.1. Animal

8–10-week-old male C57BL/6 wild-type (WT) mice (23-27 g body weight (BW)) were purchased from Shanghai SLAC Co. Ltd (Shanghai, China) and housed in an environment with controlled light (12 h light-dark cycle), temperature, and humidity, with free access to water and food. Animal protocols were approved by the Institutional Animal Care and Use Committee of Renji Hospital, School of Medicine, Shanghai Jiao Tong University.

### 2.2. Mouse Warm Liver IRI Model

An established and stable mouse model of warm liver IRI was used as we previously described [7]. Briefly, under sodium pentobarbital (40 mg/kg, i.p.) anesthesia, a midline laparotomy was performed. An atraumatic clip was placed across the portal triad, above the right lateral lobe. After 90 min of ischemia, the clamp was removed for reperfusion, animals were sacrificed after reperfusion at indicated time points, and liver tissues or serum samples were immediately collected for further analysis. Sham-operated mice underwent the same surgical procedure, but without vascular occlusion. Anesthetized mice were maintained at 37°C by means of a warming pad and heat lamp during the anesthesia process. Alda-1 (20 mg/kg BW, MCE, Monmouth Junction, NJ, USA) dissolved in solution (5% DMSO + 45% PEG400 + water) was administered intraperitoneally 2 h prior to the operation of liver ischemia, while 3-methyladenine (3MA, 30 mg/kg BW, MCE) dissolved in PBS or compound C (CC, 5 mg/kg BW, MCE) dissolved in PBS was administered intraperitoneally 1 h before the operation of liver ischemia; an equal volume of solution was administered in the same manner as the vehicle control. To distinguish the order in which the vehicle controls are administered, the vehicle controls of Alda-1 will be described as DMSO controls and the others as vehicle controls in this article.

### 2.3. Serum Sample Assays

The levels of ALT/AST in serum were measured by a standard clinical automatic analyzer (Dimension Xpand; Siemens Dade Behring, Munich, Germany). Serum TNF-*α* and IL-6 levels were measured using commercially available enzyme-linked immunosorbent assay (ELISA) kits (NeoBioscience Technology, Shenzhen, China) according to the manufacturer's protocols.

### 2.4. Liver Histopathology, Immunohistochemical, and TUNEL Staining

Standard procedures of liver histopathology, immunohistochemical, and TUNEL staining were used as we previously described [7]. Briefly, liver tissues were fixed in 4% paraformaldehyde, embedded in paraffin, and were cut into 5 *μ*m thick sections. For histopathological analysis, the sections were stained with hematoxylin and eosin (H&E), Suzuki's criteria were used to evaluate the severity of liver IRI [23], and cytoplasmic vacuolization, sinusoidal congestion, and parenchymal cell necrosis were graded from 0 to 4. For immunohistochemical staining, after deparaffinization and rehydration of the sections which were then processed for an antigen-unmasking procedure and after overnight incubation with the primary antibodies against 4HNE (rabbit polyclonal, 1 : 300, Abcam), MPO (rabbit polyclonal, 1 : 300, Abcam), cleaved caspase-3 (rabbit polyclonal, 1 : 200, Cell Signaling Technology), and F4/80 (rat monoclonal, 1 : 250, AbD Serotec) at 4°C, the sections were incubated with HRP-conjugated secondary antibodies, and immunoreactive cells were visualized using DAB. For TUNEL staining, cell death in liver paraffin sections was detected by the TUNEL method (Roche Diagnostics, Indianapolis, USA) according to the manufacturer's instructions. For each stained section, at least three images from random fields were taken, and at least three mice per group were subjected to each experiment. Image-Pro Plus software (version 6.0) was used for image analysis of the sections.

### 2.5. Isolation, Culture, and Treatment of Hepatocytes

Primary hepatocytes were isolated as we previously described [24]. For hypoxia/reoxygenation assays, hepatocytes were cultured at 37°C in a modular incubator chamber (BioSpherix, Lacona, NY, USA) gassed with a 5% CO_2_ and 95% N_2_ gas mixture for 4 h followed by 2 h reoxygenation under normoxic conditions (air/5% CO_2_). Alda-1 (20 *μ*M), 3MA (5 mM), or CC (5 *μ*M) was added to the medium for 1 hour before hypoxia. Cell viability was assessed using the CCK8 Cell Viability Assay Kit (Dojindo, Japan), and cytotoxicity was determined by lactate dehydrogenase (LDH) release (Beyotime, Shanghai, China) according to the manufacturer's instructions. All experiments were performed at least 3 times to confirm the results. Mitochondrial transmembrane potential in hepatocytes was assessed by the ΔΨ*m* assay kit with JC-1 (Beyotime) according to the manufacturer's instructions. Mitochondrial generation of superoxide was stained with MitoSOX Red (Invitrogen, Waltham, MA, USA).

### 2.6. ALDH2 Activity and MDA Content

ALDH2 activities in liver tissues were measured using an ALDH2 activity assay kit (GMS50131; GENMED, Pfizer, USA), according to the manufacturer's instruction. Briefly, the activities were measured with a spectrophotometer by monitoring the production of NADPH (340 nm). ALDH2 activity was expressed as nmol of NADH/min per mg protein (one unit). Malondialdehyde (MDA) contents were measured by commercially available kits (Nanjing Jiancheng Bioengineering Institute, Nanjing, China) according to the manufacturer's instructions.

### 2.7. Quantitative RT-PCR and Western Blot Analyses

Liver tissues and PMH were processed using RT-PCR and Western blot analysis as we previously described [24]. The relative expression levels of mRNA and protein for a target gene were normalized to relative changes with *β*-actin. The primers used for RT-PCR analysis are as follows:

- MCP1: F GGGCCTGCTGTTCACAGTT
- MCP1: R CCAGCCTACTCATTGGGAT
- IL-6: F TAGTCCTTCCTACCCCAATTTCC
- IL-6: R TTGGTCCTTAGCCACTCCTTC
- TNF-*α*: F CCCTCACACTCAGATCATCTTCT
- TNF-*α*: R GCTACGACGTGGGCTACAG
- IL-1*β*: F TGGACCTTCCAGGATGAGGACA
- IL-1*β*: R GTTCATCTCGGAGCCTGTAGTG
- *β*-Actin: F TGACAGGATGCAGAAGGAGA
- *β*-Actin: R ACCGATCCACACAGAGTACT

The primary antibodies against P62 (rabbit monoclonal, 1 : 1000, Cell Signaling Technology), cleaved caspase-3 (rabbit polyclonal, 1 : 1000, Cell Signaling Technology), Bax (rabbit polyclonal, 1 : 1000, Cell Signaling Technology), Bcl2 (rabbit monoclonal, 1 : 1000, Cell Signaling Technology), 4HNE (rabbit polyclonal, 1 : 1000, Abcam), ALDH2 (rabbit polyclonal, 1 : 3000, Proteintech), LC3B (rabbit polyclonal, 1 : 1000, Proteintech), and *β*-actin (1 : 20000, Sigma-Aldrich) were used.

### 2.8. Ultrastructure Observation of Liver Tissues by a Transmission Electron Microscope

The liver tissues were dissected, and small pieces were fixed in 2.5% glutaraldehyde. Ultrathin sections were cut and contrasted with uranyl acetate buffer by lead citrate and then observed with a Hitachi HT-7700 transmission electron microscope.

### 2.9. Statistical Analysis

All values were expressed as mean ± SEM. The unpaired Student *t*-test or one-way ANOVA test was used to compare two groups or more than two groups, respectively. For nonparametric tests, the Mann-Whitney test or Kruskal-Wallis test was used. A *P* value less than 0.05 was used to indicate a statistically significant difference in all statistical comparisons, and data were analyzed using GraphPad Prism7 (GraphPad Software Inc., San Diego, CA, USA).

## 3. Results

### 3.1. Alda-1 Pretreatment Protects Liver from IRI

**Figure 1:**
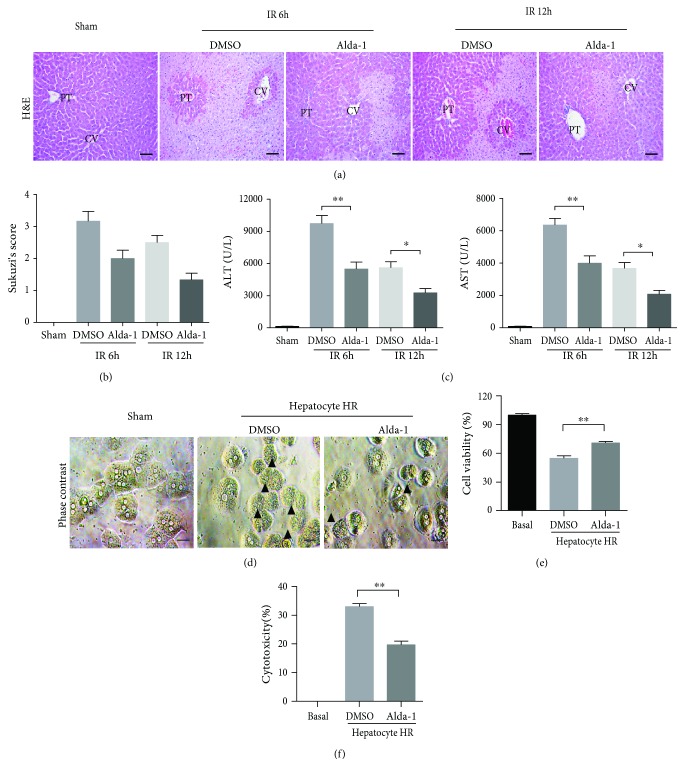
Alda-1 pretreatment protects liver from IRI. (a) Representative H&E-stained images at 6 and 12 hours after reperfusion or sham operation. PT: portal triads; CV: central veins. Scale bars: 50 *μ*m. (b) Suzuki's scores of liver sections in (a). (c) Serum ALT/AST at 6 and 12 hours after liver IRI or sham operation (*n* = 5–6 per group). (d) Visible light microphotographs were taken in primary hepatocytes after HR challenge with or without Alda-1 pretreatment. Black arrows denote damaged hepatocytes. Scale bars: 25 *μ*m. (e, f) Cell viability and cytotoxicity of primary hepatocytes after HR challenge with or without Alda-1 pretreatment were measured. Three to five independent experiments were performed. All data are shown as mean ± SEM. ^∗∗^ *P* < 0.01, ^∗^ *P* < 0.05.

To investigate whether Alda-1 has the protective effects on liver IRI, we first determined the proper dosage and time window for Alda-1 treatment in mice by measuring ALT/AST levels and found that Alda-1 treatment at 20 mg/kg and 2 h prior to liver ischemia achieved a better protection outcome (Supplementary Figures 1A and 1B). Compared to DMSO controls, mice pretreated with Alda-1 showed markedly decreased necrotic areas and Suzuki's scores at 6 h and 12 h after reperfusion (Figures 1(a) and 1(b)). Consistent with these histological alterations, Alda-1-pretreated mice also exhibited lower serum ALT/AST levels (Figure 1(c)). To detect the function of Alda-1 on primary hepatocytes, we adopted the hypoxia/reoxygenation (H/R) model to treat primary hepatocytes *in vitro*. Compared to DMSO controls, the primary hepatocyte treated with Alda-1 showed fewer morphology abnormalities, higher cell viability, and lower cytotoxicity (Figures 1(d)–1(f)). Collectively, these findings demonstrated that Alda-1 had a protective role in liver IRI.

### 3.2. Alda-1 Protects Hepatocytes from IR-Induced Apoptosis

**Figure 2:**
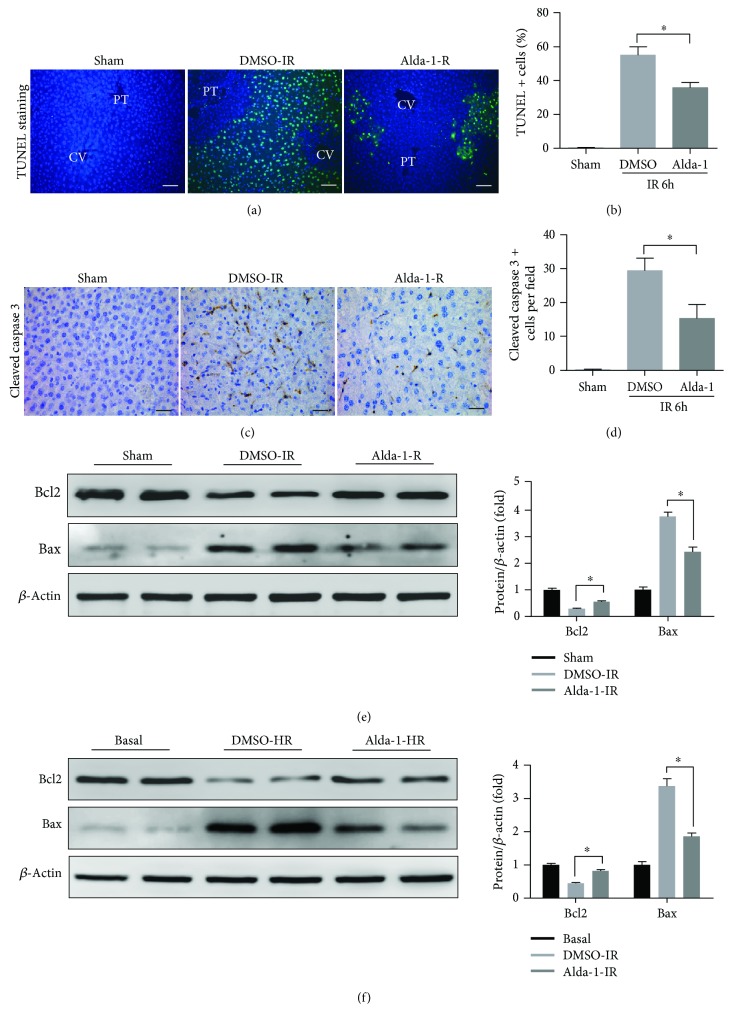
Alda-1 pretreatment alleviates apoptosis in liver IRI both in vivo and in vitro. (a, b) Representative sections of TUNEL staining and the numbers of TUNEL-positive cells in liver sections at 6 h after reperfusion or sham operation. Scale bars: 50 *μ*m. (c, d) Representative sections of cleaved caspase-3 staining and the number of cleaved caspase-3-positive cells at 6 h after reperfusion or sham operation. Scale bars: 25 *μ*m. (e) Western blot analysis of Bcl2 and Bax expression in liver tissues at 6 hours after reperfusion or sham operation (*β*-actin is used as a loading control). (f) Western blot analysis of Bcl2 and Bax expression in primary hepatocytes after HR challenge (*β*-actin is used as a loading control). All data are shown as mean ± SEM (*n* = 4–6). ^∗^ *P* < 0.05.

Apoptosis is a crucial mechanism to induce hepatocyte death in the process of liver IRI [25]. We then examined hepatic apoptosis by measuring levels of TUNEL, cleaved caspase-3, Bax, and Bcl2. Compared to DMSO controls, mice pretreated with Alda-1 showed significantly reduced hepatic apoptosis levels as evidenced by decreased TUNEL, cleaved caspase-3, and Bax signals and increased Bcl2 levels (Figures 2(a)–2(e)). In addition, Alda-1 treatment caused an increase in Bcl2 and a decrease in Bax levels in primary hepatocytes subjected to H/R challenge *in vitro* (Figure 2(f)). These findings demonstrated that Alda-1 could reduce hepatic apoptosis during liver IRI.

### 3.3. Alda-1 Ameliorates Inflammatory Responses in Liver IRI

**Figure 3:**
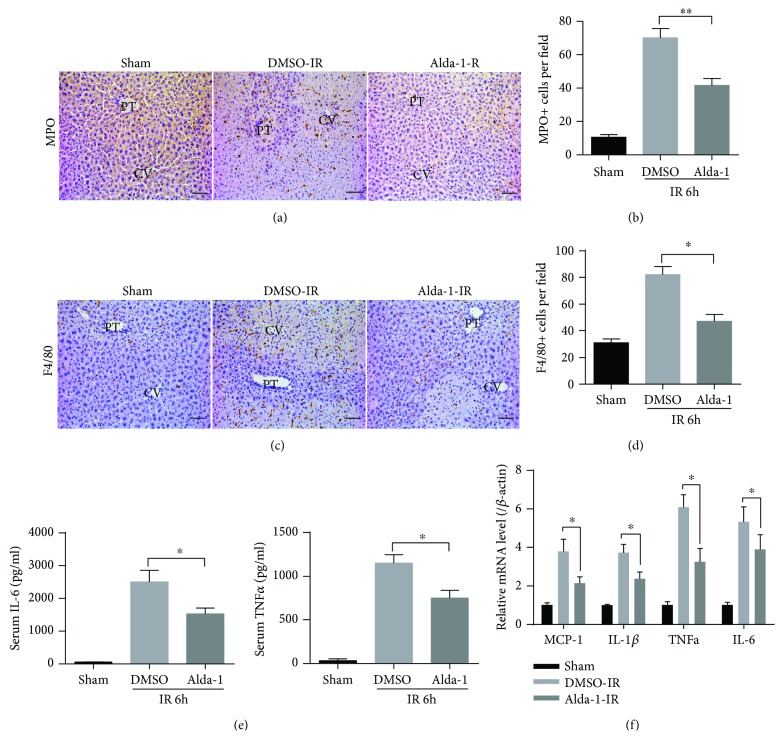
Alda-1 pretreatment restrains inflammatory responses in livers during IR injury. (a, b) Representative sections of MPO staining and the numbers of MPO-positive cells in liver sections at 6 h after reperfusion or sham operation. PT: portal triads; CV: central veins. Scale bars: 50 *μ*m. (c, d) Representative sections of F4/80 staining and the numbers of F4/80-positive cells in liver sections at 6 h after reperfusion or sham operation. PT: portal triads; CV: central veins. Scale bars: 50 *μ*m. (e) Serum IL-6 and TNF-*α* levels at 6 h after reperfusion or sham operation were measured by ELISA. (f) The mRNA levels of cytokines and chemokines at 6 h after reperfusion or sham operation were determined by quantitative RT-PCR. All data are shown as mean ± SEM (*n* = 4–6). ^∗∗^ *P* < 0.01, ^∗^ *P* < 0.05.

During liver IRI, hepatocyte death-induced inflammatory responses can further exacerbate the severity of liver IRI [26]. We then sought to assess whether Alda-1 also affects inflammatory responses after liver IRI. As shown in Figures 3(a)–3(d), the hepatic infiltration of neutrophil (MPO) and macrophage (F4/80) was significantly decreased in Alda-1-pretreated mice compared to DMSO controls. In addition, both serum and hepatic levels of proinflammatory cytokines and chemokines such as MCP-1, IL-1*β*, TNF-*α*, and IL-6 were lower in Alda-1-pretreated mice than in control mice (Figures 3(e) and 3(f)). Collectively, these findings manifested that Alda-1 decreased inflammatory responses in liver IRI.

### 3.4. Alda-1 Administration Reduces Mitochondrial Dysfunction and ROS Production after H/R Challenge *In Vitro*

**Figure 4:**
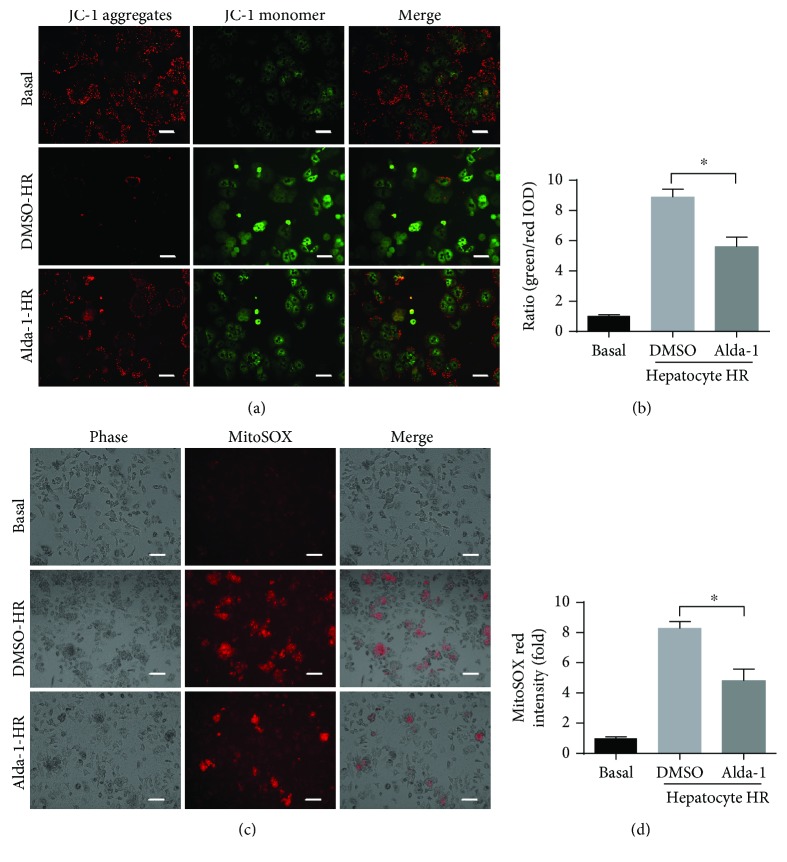
Alda-1 pretreatment alleviates mitochondrial injury and ROS production after HR challenge in vitro. (a, b) Representative pictures of mitochondrial membrane potential of primary hepatocytes after HR challenge with or without Alda-1 pretreatment. The ratio of green to red fluorescence intensity was determined. Scale bars: 25 *μ*m. (c, d) Representative pictures of mitochondrial ROS accumulation of primary hepatocytes after HR injury. The mitochondrial ROS was expressed by the relative red area of the total picture. Scale bars: 50 *μ*m. Data are shown as mean ± SEM (*n* = 4–6). ^∗^ *P* < 0.05.

At the cellular level, mitochondrial dysfunction and excessive ROS production are two initial detrimental events to drive hepatocyte death and inflammatory responses during liver IRI [10]. To investigate whether Alda-1-mediated hepatoprotection during IRI is due to improved mitochondrial dysfunction and ROS production, we measured the mitochondrial membrane potential and ROS levels in primary hepatocytes during H/R treatment *in vitro*. Compared with the DMSO controls, Alda-1 significantly prevented the decrease in mitochondrial membrane potential (using JC-1 fluorescent dye) and the increase in ROS production (using mitoSOX Red dye) during H/R challenge (Figures 4(a)–4(d)). Thus, we concluded that Alda-1 pretreatment could protect against mitochondrial dysfunction and inhibit oxidative stress during liver IRI.

### 3.5. Alda-1 Pretreatment Enhances ALDH2 Activity and Decreases Toxic Aldehydes during Liver IRI

**Figure 5:**
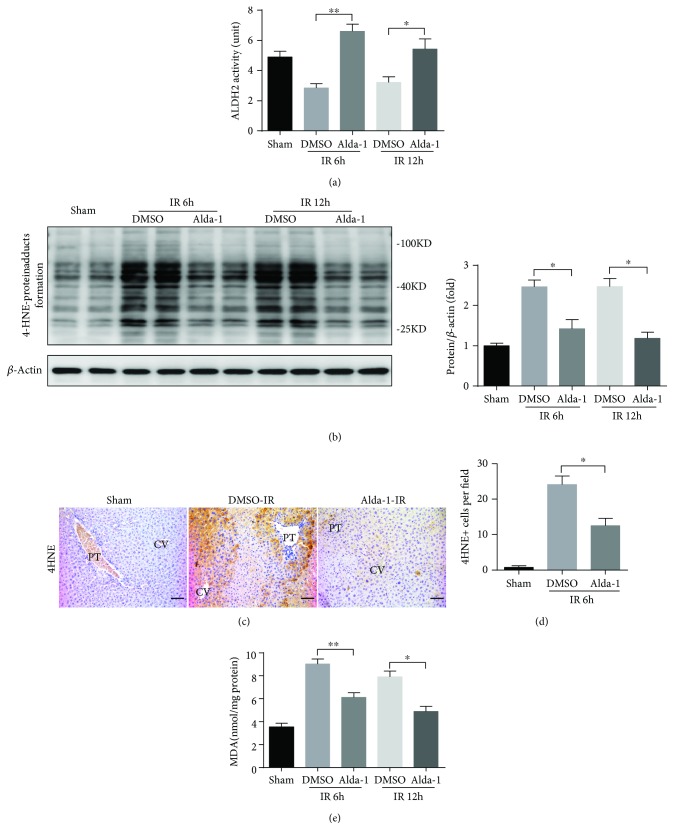
Alda-1 has the efficacy of ALDH2 activity activation and toxic aldehyde clearance during liver IR. (a) ALDH2 activity was observed in liver tissues at 6 and 12 hours after reperfusion or sham operation (*n* = 4–6). (b) Western blot analysis of 4HNE protein adduct expression in liver tissues at 6 and 12 hours after reperfusion or sham operation (*β*-actin is used as a loading control). (c, d) Representative sections of 4HNE staining and the numbers of 4HNE-positive cells in liver sections at 6 h after reperfusion or sham. PT: portal triads; CV: central veins. Scale bars: 50 *μ*m. (e) The malondialdehyde (MDA) content was measured in liver tissues at 6 and 12 hours after reperfusion or sham operation. All data are shown as mean ± SEM (*n* = 4–6). ^∗∗^ *P* < 0.01, ^∗^ *P* < 0.05.

As Alda-1 is an ALDH2 activator, we then examined whether Alda-1 treatment activates ALDH2. During liver IRI, although ALDH2 activity was significantly decreased at 6 h and 12 h after reperfusion, Alda-1 pretreatment markedly increased hepatic ALDH2 activity (Figure 5(a)). Interestingly, such ALDH2 activity changes have nothing to do with ALDH2 expression because Western blot assays showed that there were no ALDH2 protein level changes in either sham mice, vehicle controls, or Alda-1 pretreated mice (Supplementary Figures 2A and 2B). As ALDH2 is a key enzyme responsible for the removal of reactive aldehydes, we then investigated whether Alda-1 pretreatment could reduce the accumulation of reactive aldehydes during liver IRI. Both Western blot and IHC assays showed that compared to the vehicle control, Alda-1 pretreatment significantly reduced the levels of 4HNE adducts at 6 h or 12 h after liver reperfusion (Figures 5(b)–5(d)). In addition, Alda-1 pretreatment also prevented the accumulation of MDA, another type of reactive aldehyde, at 6 h and 12 h after reperfusion (Figure 5(e)). Therefore, these results demonstrated that Alda-1 pretreatment could increase ALDH2 activity and in turn scavenge reactive aldehydes.

### 3.6. Alda-1-Mediated Hepatoprotection during Liver IRI Is Dependent on Enhanced Autophagy

**Figure 6:**
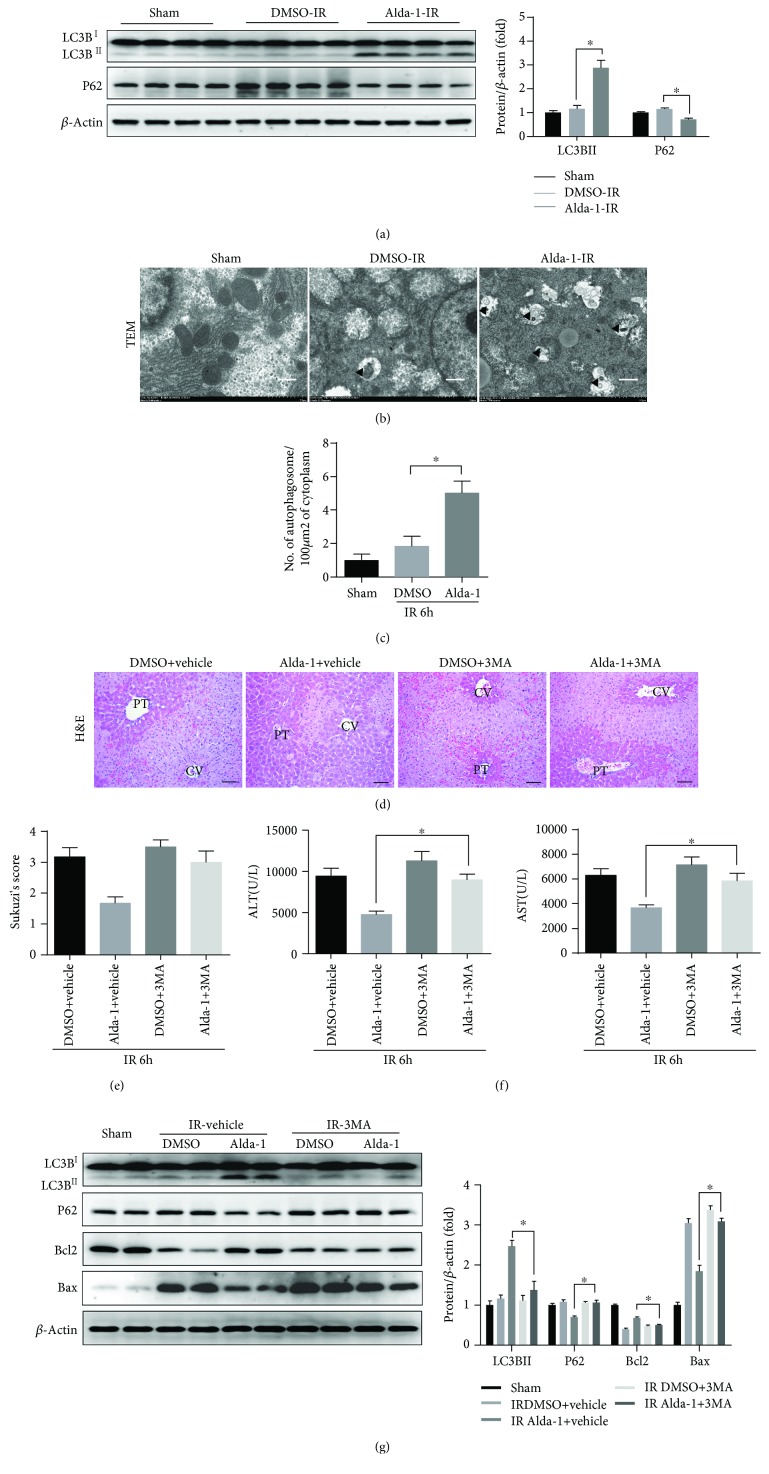
Autophagy is involved in ALDH2 activation-induced protection of mouse liver IRI. (a) Western blot analysis of LC3B and P62 expression in liver tissues at 6 hours after reperfusion or sham operation (*β*-actin is used as a loading control). (b, c) Representative transmission electron micrographs showing autophagosomes in the ischemic liver tissues at 6 h after reperfusion (black arrows denote autophagosomes) and the number of autophagosomes in per 100 *μ*m^2^ of the cytoplasm. PT: portal triads; CV: central veins. Scale bars: 50 *μ*m. Scale bars: 1 *μ*m. (d–g) Mice were treated with 3-methyladenine 1 h after Alda-1 or pretreated with DMSO and killed at 6 h after reperfusion. (d, e) Representative H&E-stained images and relative Suzuki's scores of the liver section. Scale bars: 50 *μ*m. (f) Serum ALT/AST level. (g) Western blot analysis of LC3B, P62, Bcl2, and Bax expression in liver tissues (*β*-actin is used as a loading control). All data are shown as mean ± SEM (*n* = 4–6). ^∗^ *P* < 0.05.

Previous studies have demonstrated that ALDH2 activation is sufficient to induce autophagy in different physiological and pathological conditions [27, 28]. Consistent with those findings, compared to DMSO controls, Alda-1 treatment significantly enhanced liver autophagy as indicated by increased LC3BII and decreased P62 levels (Figure 6(a) and Supplementary Figure 3A). Correspondingly, the number of autophagosomes in the liver of Alda-1-pretreated mice was considerably increased (Figures 6(b) and 6(c)). To test whether Alda-1-mediated hepatoprotection was dependent on the enhanced autophagy, we investigated the effect of Alda-1 by blocking autophagy with 3-methyladenine (3MA, an autophagy inhibitor). H&E staining and Suzuki's scores showed that the necrotic areas in the liver of Alda-1-pretreated mice were significantly decreased compared to those of the DMSO controls, whereas inhibition of autophagy reversed the protective effect of Alda-1 (Figures 6(d) and 6(e)). In line with the histological data, 3MA-mediated autophagy inhibition also reversed the serum ALT/AST level decrease in Alda-1-treated mice (Figure 6(f)). Western blot assays showed that 3MA abrogated the Alda-1-induced expression increases in LC3BII and Bcl2 and decreases in P62 and Bax (Figure 6(g)). In addition, 3MA-mediated autophagy inhibition sensitize Alda-1-treated primary hepatocytes to H/R injury *in vitro* as indicated by decreased cell viability and increased LDH release levels (Supplementary Figures 3B and 3C). As we have previously demonstrated that rapamycin treatment protects the liver from IRI via both autophagy induction and mTORC2-Akt activation [29], we then test whether rapamycin treatment could further potentiate the hepatoprotective effects of Alda-1. We found that although either Alda-1 or rapamycin treatment decreased liver necrosis and serum ALT/ALT levels, no synergistic effects were observed in the case of cotreatment of Alda-1 and rapamycin (Supplementary Figures 4A–4C). Collectively, these results indicated that Alda-1 pretreatment plays a protective role in liver IRI through the enhancement of autophagy.

### 3.7. Alda-1-Induced Autophagy Enhancement during Liver IRI Is Mediated by AMPK Activation

**Figure 7:**
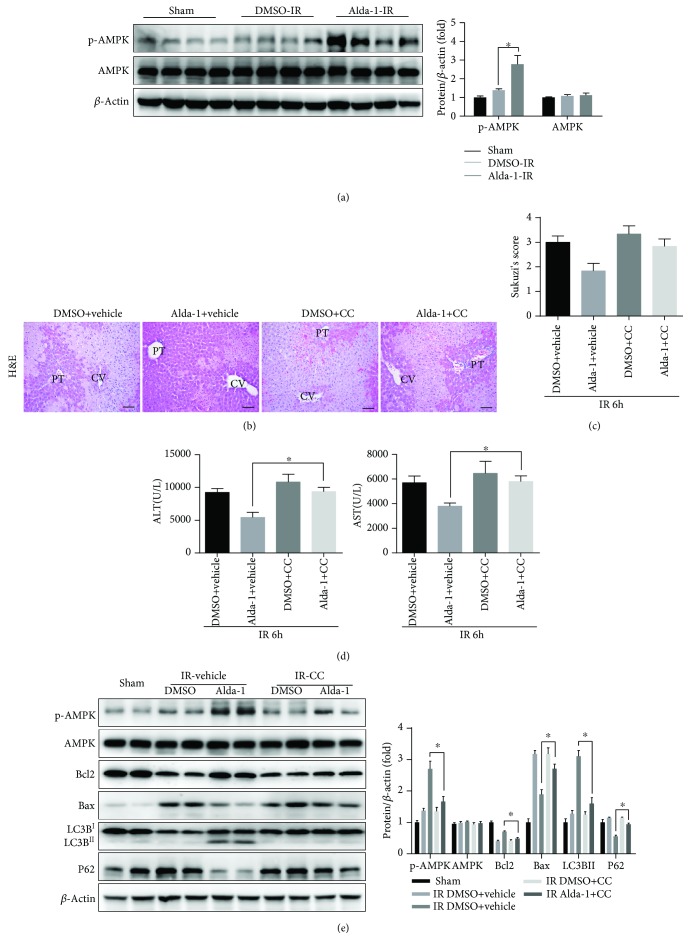
AMPK activation is involved in autophagy enhancement by Alda-1 pretreatment during liver IR. (a) Western blot analysis of p-AMPK and AMPK expression in liver tissues at 6 hours after reperfusion or sham operation (*β*-actin is used as a loading control). (b–d) Mice were treated with CC 1 h after Alda-1 or pretreated with DMSO and killed at 6 h after reperfusion. (b, c) Representative H&E-stained images and relative Suzuki's scores of the liver section. PT: portal triads; CV: central veins. Scale bars: 50 *μ*m. Scale bars: 50 *μ*m. (d) Serum ALT/AST level. (e) Western blot analysis of p-AMPK, AMPK, Bcl2 and Bax, LC3B, and P62 expression in liver tissues (*β*-actin is used as a loading control). All data are shown as mean ± SEM (*n* = 4–6). ^∗^ *P* < 0.05.

Previous studies have shown that reactive aldehydes such as 4HNE impair the activation of AMPK signaling [30, 31]. Given that AMPK activation plays a protective role in liver IRI at least partly through the activation of the autophagy pathway [32, 33], we then tested whether the Alda-1-mediated hepatoprotection during liver IRI is attributed to AMPK activation. Western blot assays showed that, compared to sham and vehicle controls, Alda-1 pretreatment markedly increased the phosphorylation levels of AMPK in liver tissues after IRI (Figure 7(a)). Consistently, Alda-1 treatment also enhanced AMPK phosphorylation in primary hepatocytes after H/R challenge (Supplementary Figure 5A). To determine whether Alda-1-induced autophagy enhancement is dependent on AMPK activation, compound C (CC, a specific AMPK inhibitor) was employed to block AMPK activation during liver IRI. AMPK inhibition by CC blunted the hepatoprotection of Alda-1 during liver IRI as evidenced by increased hepatic necrosis areas and serum ALT/AST levels (Figures 7(b)–7(d)). In addition, Western blot showed that CC treatment resulted in a significant decrease in AMPK phosphorylation and Bcl2 and LC3BII expression and a marked increase in P62 and Bax expression (Figure 7(e)). Correspondingly, CC treatment significantly decreased cell viability and increased LDH release in primary hepatocytes treated with Alda-1 after H/R *in vitro* (Supplementary Figures 5B and 5C). Thus, these findings suggested that AMPK activation is necessary for Alda-1-mediated autophagy activation during liver IRI.

## 4. Discussion

In our present study, we demonstrated that Alda-1, an ALDH2 agonist, protects against liver IRI. In detail, Alda-1 treatment attenuated liver necrosis and hepatocyte apoptosis, reduced inflammatory responses, and inhibited mitochondrial dysfunction and ROS production during liver IRI. The underlying mechanisms of the protective role of Alda-1 were associated with the direct clearance of reactive aldehydes and the indirect autophagy enhancement which is induced by AMPK activation.

During IRI, increased ROS production is a major detrimental event to cause cell damage and even death, partly because ROS attacks various critical biological lipids, particularly membrane phospholipids, leading to the formation of reactive aldehydes, such as 4HNE and MDA, to further aggravate the injury [34]. Although multiple lines of evidence has demonstrated that ALDH2 is the major enzyme to detoxify those reactive aldehydes, the phenomenon that ALDH2 activity is usually inhibited during IRI makes it impossible to scavenge reactive aldehydes effectively [15, 16, 19–22], which consequently leads to massive accumulation of those toxic reactive aldehydes and cell damage. Therefore, activating ALDH2 is a conceivable approach to improve IRI. In fact, administration of Alda-1 has been demonstrated to improve IRI in many other organs except the liver [16, 19–22]. In the present study, we also demonstrated decreased ALDH2 activity in the mouse liver IRI model and administration of Alda-1 could significantly increase ALDH2 activity, which was independent of ALDH2 expression changes. Consequently, Alda-1-mediated enhancement of ALDH2 activity blocked the accumulation of 4HNE and MDA and improved the liver IRI.

In addition to causing cell damage and death, reactive aldehydes have also been reported to activate the NF-*κ*B pathway linking to activation of inflammatory responses [35]; therefore, reactive aldehydes could induce inflammatory responses directly and indirectly. In the present study, we show that Alda-1 treatment could ameliorate hepatocyte apoptosis and sterile inflammation during liver IRI, which is consistent with previous reports showing that Alda-1 has both antiapoptosis and anti-inflammatory properties [19–22].

Autophagy is generally recognized as a cellular protective pathway in response to various intracellular or extracellular stimuli. Although the function of autophagy in IRI remains controversial, we and other groups have identified the protective role of autophagy during liver IRI [7–9, 29]. In the present study, we found autophagy enhancement after Alda-1 treatment with increased levels of LC3BII, P62 degradation, and autophagosomes. Apparently, the hepatoprotective role of Alda-1 was dependent on autophagy because 3MA-mediated autophagy inhibition greatly diminished the hepatoprotective effects of Alda-1 during liver IRI or *in vitro* H/R treatment. Interestingly, rapamycin-induced autophagy enhancement could not further augment Alda-1-mediated hepatoprotection, suggesting that Alda-1 and rapamycin do not work synergistically.

A recent study has shown that protein adducts of reactive aldehydes inhibit the activation of LKB resulting in impaired signaling activity of the LKB1/AMPK/mTOR pathway [30]. In addition, ALDH2 has been reported to protect myocardial function through an AMPK-dependent autophagy activation axis in an experimental diabetic cardiomyopathy model [28]. In the present study, we also found that Alda-1 treatment induced the activation of AMPK, whereas compound C-mediated AMPK inhibition greatly abrogated the protective role of Alda-1 and autophagy activation. Moreover, given the fact that 4HNE could directly target AMPK to inhibit its activity [31], we propose that Alda-1 treatment directly or indirectly activates AMPK resulting in autophagy activation during liver IRI.

## Data Availability

The data used to support the findings of this study are available from the corresponding author upon request.

## Supplementary Materials

Supplementary Materials Supplementary Figure 1: Alda-1 usage at different concentrations and times. Supplementary Figure 2: the expression of ALDH2 in liver IR. Supplementary Figure 3: autophagy enhancement in hepatocyte HR challenge. Supplementary Figure 4: combined use of rapamycin and Alda-1. Supplementary Figure 5: AMPK activation in hepatocyte HR challenge.
