# Supplementary material for: Alda-1 Ameliorates Liver Ischemia-Reperfusion Injury by Activating Aldehyde Dehydrogenase 2 and Enhancing Autophagy in Mice
Source: J Immunol Res. 2018 Dec 24;2018:9807139. doi: 10.1155/2018/9807139 (PMC6323494; doi:10.1155/2018/9807139)

Supplemental data

Meng Li et al **Alda-1 Ameliorates Mouse Liver Ischemia-Reperfusion Injury by Activating Aldehyde Dehydrogenase 2 and Enhancing Autophagy**

MATERIALS AND METHODS

Isolation of mitochondria and cytosol fractions, western blots

Mitochondrial and cytosolic fractions of liver tissue were prepared by using a mitochondrial isolation kit (Beyotime, Shanghai, China) according to the manufacturer's protocol. The primary antibodies against ALDH2, COX IV were used.

Chemical compounds

Rapamycin (RPM, 5mg/kg BW, MCE, Monmouth Junction, NJ, USA) which dissolved in vehicle (2% DMSO+30% PEG 400+5% Tween 80+ddH2O) was administered intraperitoneally 1h after the use of Alda-1 or DMSO, about 1h prior to the onset of liver ischemia.

Supplementary Figure legends

Supplementary Figure .1 : Liver IR and Alda-1 pretreatment don't influence the protein level of ALDH2. (A)Western blot analysis of ALDH2 expression in liver tissues at 6 hours after reperfusion or Sham operation (β-actin is used as a loading control). Data shown as mean±S.E.M.(n=4). (B)Western blot analysis of ALDH2 expression in cytosolic and mitochondrial fractions (β-actin and COX IV are used as loading control for cytosol and mitochondria).

Supplementary Figure .2 : Autophagy were enhanced during Alda-1 treatment in HR model, and autophagy inhibitor could impede the protection. (A) Western blot analysis of LC3B and P62 expression in cultured primary hepatocytes after HR challenge (*β*-actin is used as a loading control).(B,C) Cell viability and cytotoxicity of primary hepatocytes after HR challenge with 3-MA in the presence and absence of Alda-1 pretreatment were measured. Three to five independent experiments were performed. All data shown as mean±S.E.M.. *$P<0.05$.

Supplementary Figure .3 : AMPK inhibitor could reverse the effect of Alda-1 in HR model. (A) Western blot analysis of p-AMPK and AMPK expression in cultured primary hepatocytes after HR challenge (*β*-actin is used as a loading control).(B,C) Cell viability and cytotoxicity of primary hepatocytes after HR challenge with CC in the presence and absence of Alda-1 pretreatment were measured. Three to five independent experiments were performed. All data shown as mean±S.E.M.. *$P<0.05$.

Supplementary Figure .4: Combined use Rapamycin slightly potentiate the effects of Alda-1. Mice were treated with Rapamycin 1h after Alda-1 or DMSO

pretreatment and killed at 6 h after reperfusion. (A, B) Representative H&E-stained images and the relative Suzuki's scores in liver section. PT: portal triads. CV: central veins. Scale bars: 50μm. Scale bars: 50μm. (C) Serum ALT/AST level. All data shown as mean±S.E.M.(n=4-6).

Supplementary Figure .5: The usage of different doses and time of Alda-1. (A) Mice were treated with different doses of Alda-1 or DMSO 2h prior to the operation of liver ischemia and killed at 6 h after reperfusion. ALT and AST were measured in serum. (B) Mice were treated with Alda-1 2h pre-operation, at the onset of ischemia or 2h post-operation and killed at 6 h after reperfusion. ALT and AST were measured in serum. All data shown as mean±S.E.M.(n=4-5).

Supplementary Figure 1

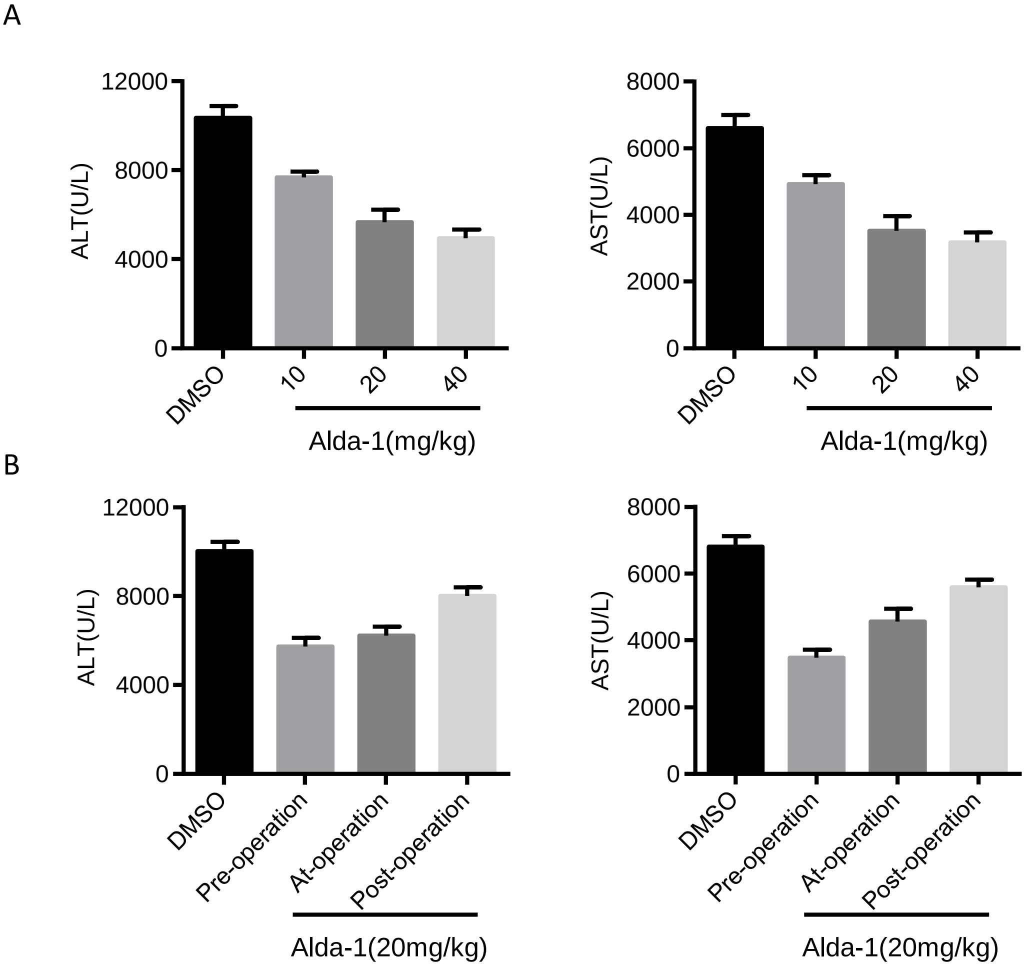

Supplementary Figure 2

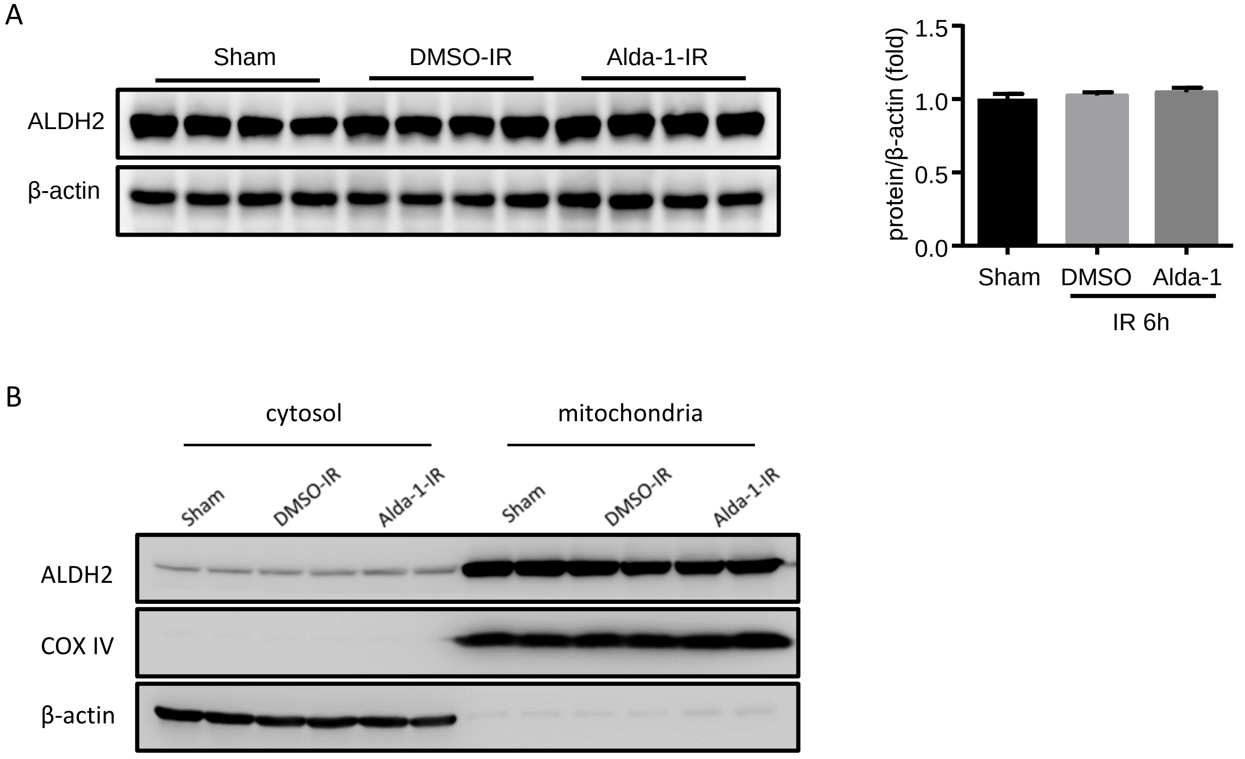

Supplementary Figure 3

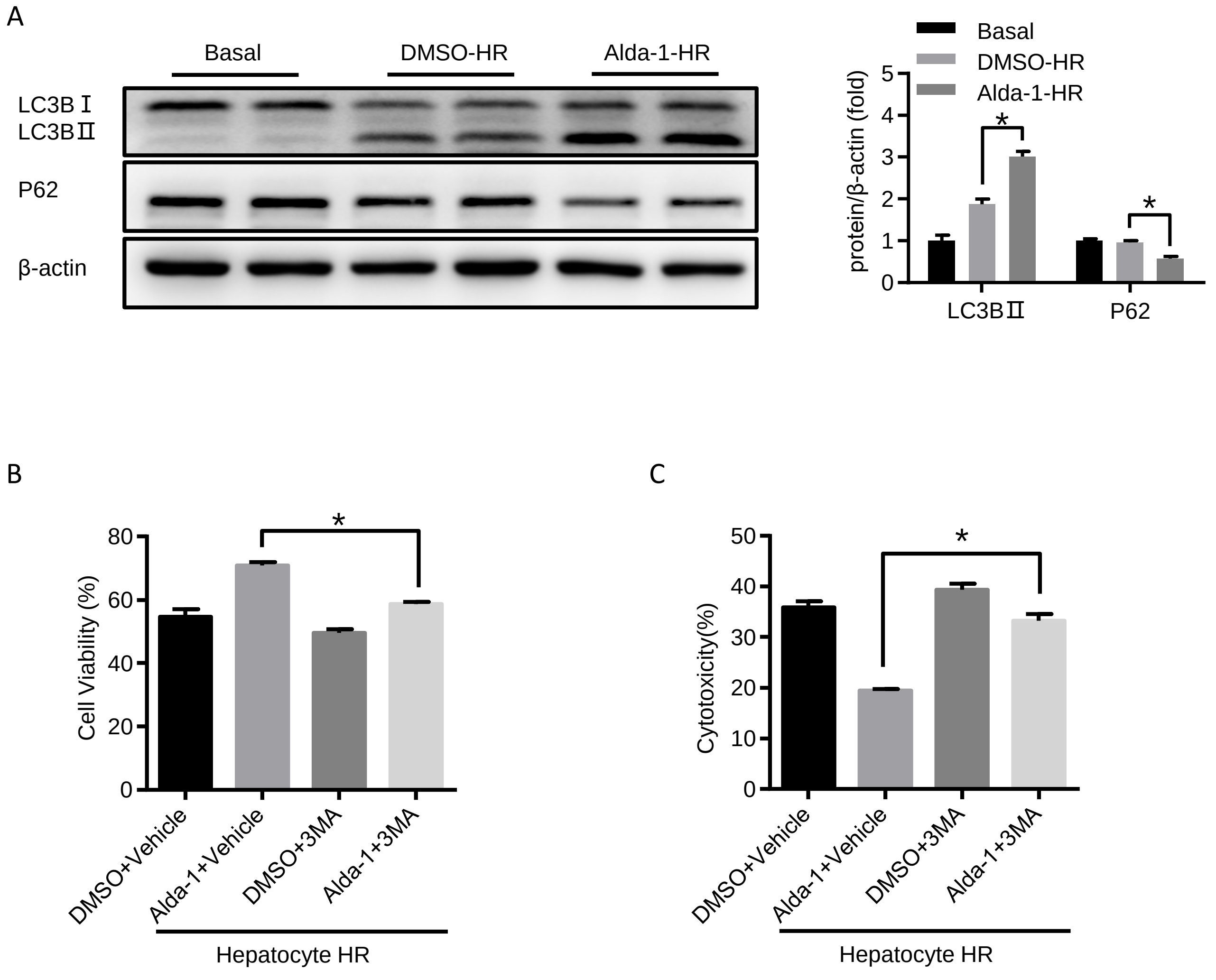

Supplementary Figure 4

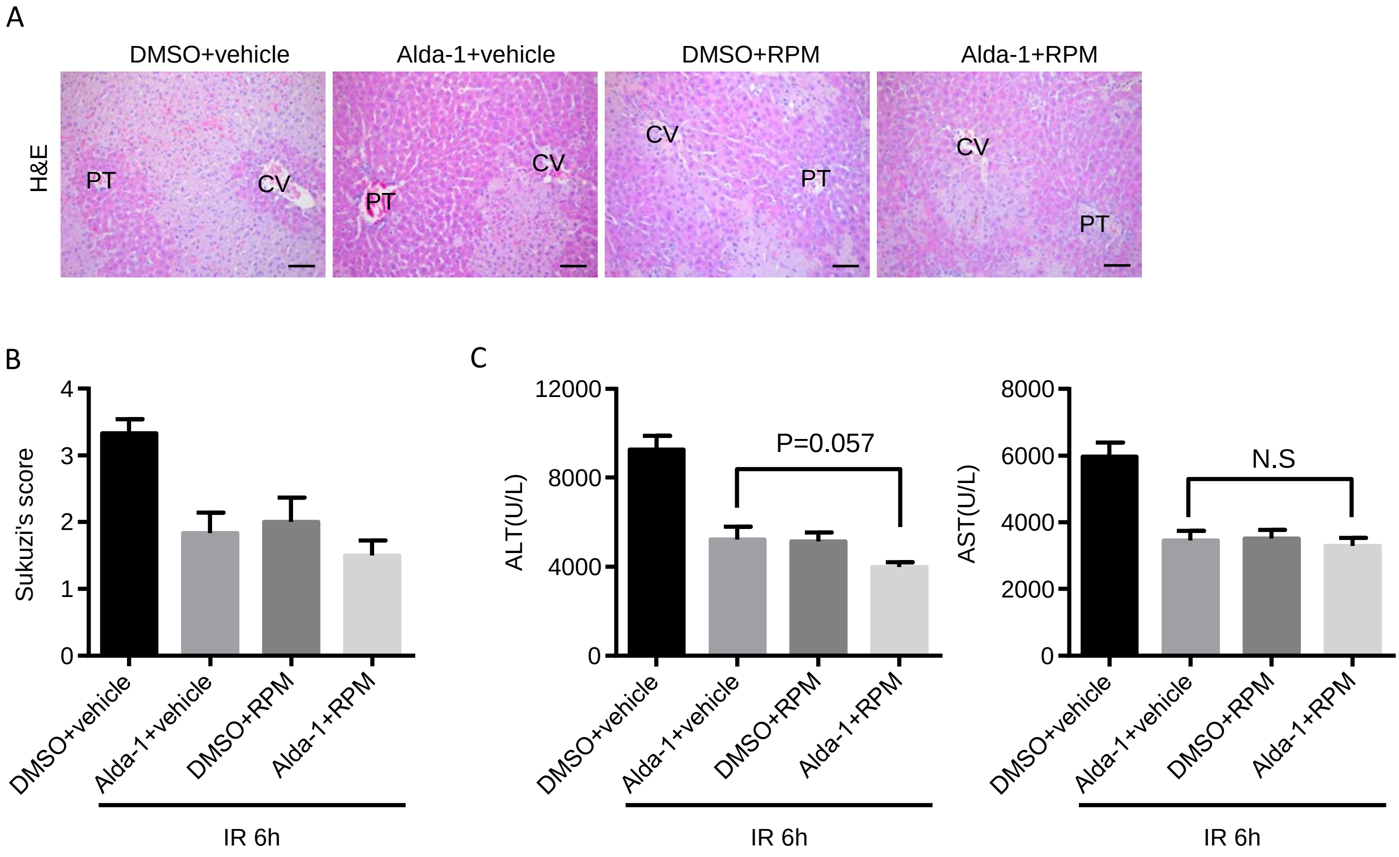

Supplementary Figure 5

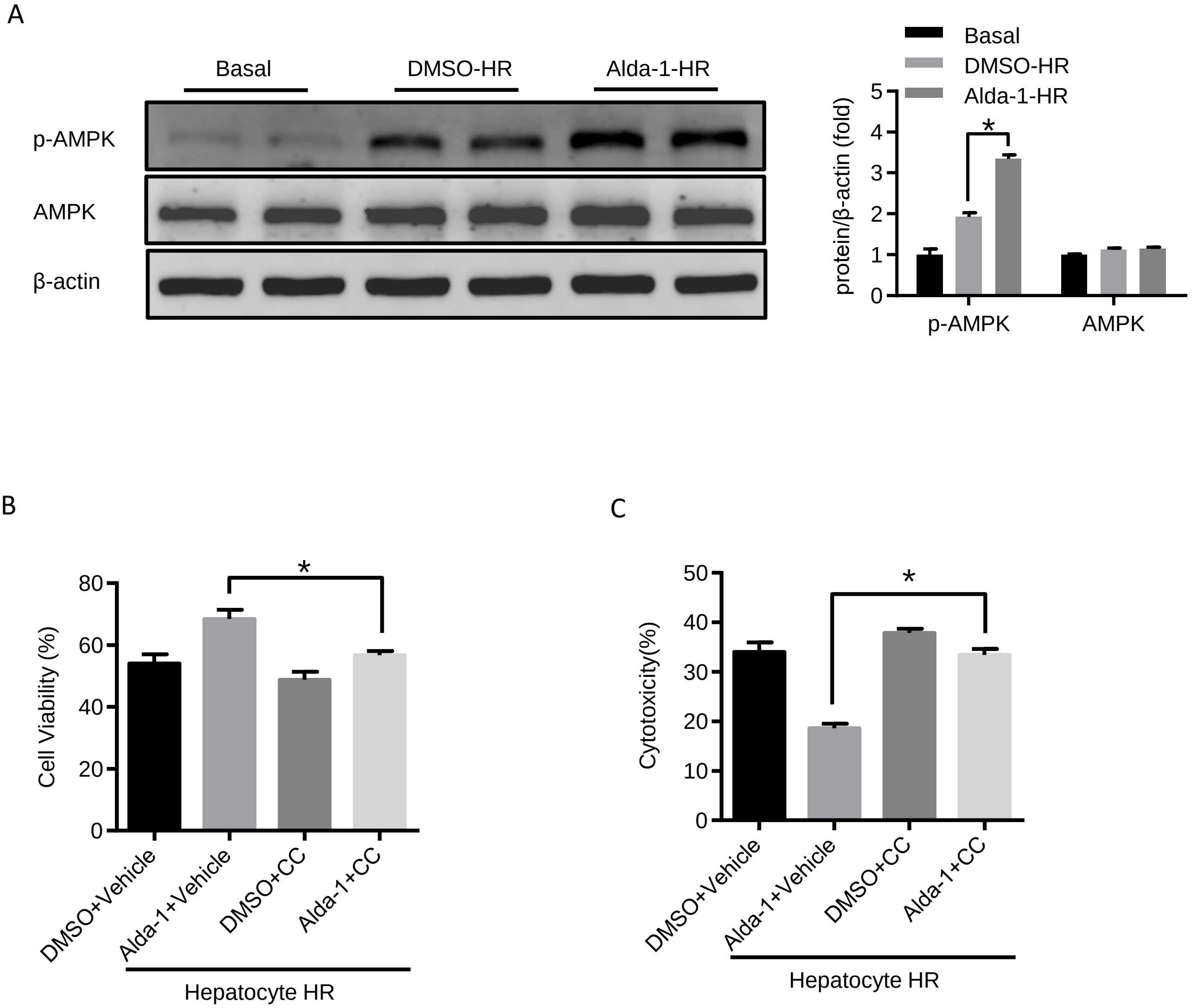

Supplement: Supplementary Materials — Supplementary Figure 1: Alda-1 usage at different concentrations and times. Supplementary Figure 2: the expression of ALDH2 in liver IR. Supplementary Figure 3: autophagy enhancement in hepatocyte HR challenge. Supplementary Figure 4: combined use of rapamycin and Alda-1. Supplementary Figure 5: AMPK activation in hepatocyte HR challenge. [file 9807139.f1.pdf]
